# Hybrid promoter engineering strategies in *Yarrowia lipolytica*: isoamyl alcohol production as a test study

**DOI:** 10.1186/s13068-021-02002-z

**Published:** 2021-07-02

**Authors:** Yu Zhao, Shiqi Liu, Zhihui Lu, Baixiang Zhao, Shuhui Wang, Cuiying Zhang, Dongguang Xiao, Jee Loon Foo, Aiqun Yu

**Affiliations:** 1 State Key Laboratory of Food Nutrition and Safety, Key Laboratory of Industrial Fermentation Microbiology of the Ministry of Education, Tianjin Key Laboratory of Industrial Microbiology, College of Biotechnology, Tianjin University of Science and Technology, No. 29 the 13th Street TEDA, Tianjin, 300457 People’s Republic of China; 2 Synthetic Biology Translational Research Programme, Yong Loo Lin School of Medicine, National University of Singapore, Singapore, 119228 Singapore; 3 NUS Synthetic Biology for Clinical and Technological Innovation (SynCTI), National University of Singapore, Singapore, 117456 Singapore; 4 Department of Biochemistry, Yong Loo Lin School of Medicine, National University of Singapore, Singapore, 117597 Singapore

**Keywords:** *Y. lipolytica*, Metabolic engineering, Hybrid promoter, Isoamyl alcohol, Synthetic promoter

## Abstract

**Background:**

In biological cells, promoters drive gene expression by specific binding of RNA polymerase. They determine the starting position, timing and level of gene expression. Therefore, rational fine-tuning of promoters to regulate the expression levels of target genes for optimizing biosynthetic pathways in metabolic engineering has recently become an active area of research.

**Results:**

In this study, we systematically detected and characterized the common promoter elements in the unconventional yeast *Yarrowia lipolytica*, and constructed an artificial hybrid promoter library that covers a wide range of promoter strength. The results indicate that the hybrid promoter strength can be fine-tuned by promoter elements, namely, upstream activation sequences (UAS), TATA box and core promoter. Notably, the UASs of *Saccharomyces cerevisiae* promoters were reported for the first time to be functionally transferred to *Y. lipolytica*. Subsequently, using the production of a versatile platform chemical isoamyl alcohol as a test study, the hybrid promoter library was applied to optimize the biosynthesis pathway expression in *Y. lipolytica*. By expressing the key pathway gene, *ScARO10*, with the promoter library, 1.1–30.3 folds increase in the isoamyl alcohol titer over that of the control strain *Y. lipolytica* Po1g *KU70*∆ was achieved. Interestingly, the highest titer increase was attained with a weak promoter P_*UAS1B4-EXPm*_ to express *ScARO10*. These results suggest that our hybrid promoter library can be a powerful toolkit for identifying optimum promoters for expressing metabolic pathways in *Y. lipolytica*.

**Conclusion:**

We envision that this promoter engineering strategy and the rationally engineered promoters constructed in this study could also be extended to other non-model fungi for strain improvement.

**Supplementary Information:**

The online version contains supplementary material available at 10.1186/s13068-021-02002-z.

## Background

Promoters are one of the most important components in synthetic biology, and well-controlled promoters are very critical for regulating gene expression in eukaryotes. The activity of a promoter is co-regulated by various elements. In yeast, the common promoter elements usually include upstream activation sequences (UAS), TATA box and core promoter [1, 2]. By rational modification of these elements, the activity of promoters can be fine-tuned.

At the beginning of transcription, regulatory signals are transmitted from the UAS to the core promoter, the site where the transcription factors and the RNA polymerase II assembles to form the transcription preinitiation complex (PIC) [3]. The core promoter significantly contributes to the regulation of gene expression and is also the key factor determining the promoter strength. Although core promoters were initially thought to be invariant, researchers have found that they exhibit great structural and functional diversity [4, 5]. TATA box, the recognition site of the transcription factor TATA binding protein (TBP), is one of the first kind of functional elements identified to regulate the promoter strength of the core promoter and typically located 40–120 bp upstream of the transcription start site (TSS) [3, 6]. Mutations in the TATA box usually alter the promoter strength [1, 7–10]. The UAS, which is usually located at the 5′ end of the promoter [3, 11], is also known to have an impact on the strength of the promoter by varying its copy number. By analysing the *Yarrowia lipolytica* alkaline extracellular protease 2 (*XPR2*) gene promoter, P_*XPR2*_, Madzak et al. identified UAS1B to be the most significant functional element that activates the promoter [12]. Subsequently, evaluation of a hybrid promoter library consisting of a minimal P_*LEU*_ fragment and different copy numbers of the UAS1B indicates that enhancement in promoter strength is correlated to increased copy number of UAS1B [12]. When present in a promoter, distinct types of UAS can cooperate to control transcription. For example, by combining different UAS elements (UASTEF and UAS1B) in *Y. lipolytica*, the expression level of a constructed promoter was sevenfold higher than the wild-type promoter [2]. Taken together, by exploring the synergy between various promoter elements and understanding the working mechanism of the promoter, promoters with stronger activity and a wider range of expression levels can be constructed.

**Fig. 1:**
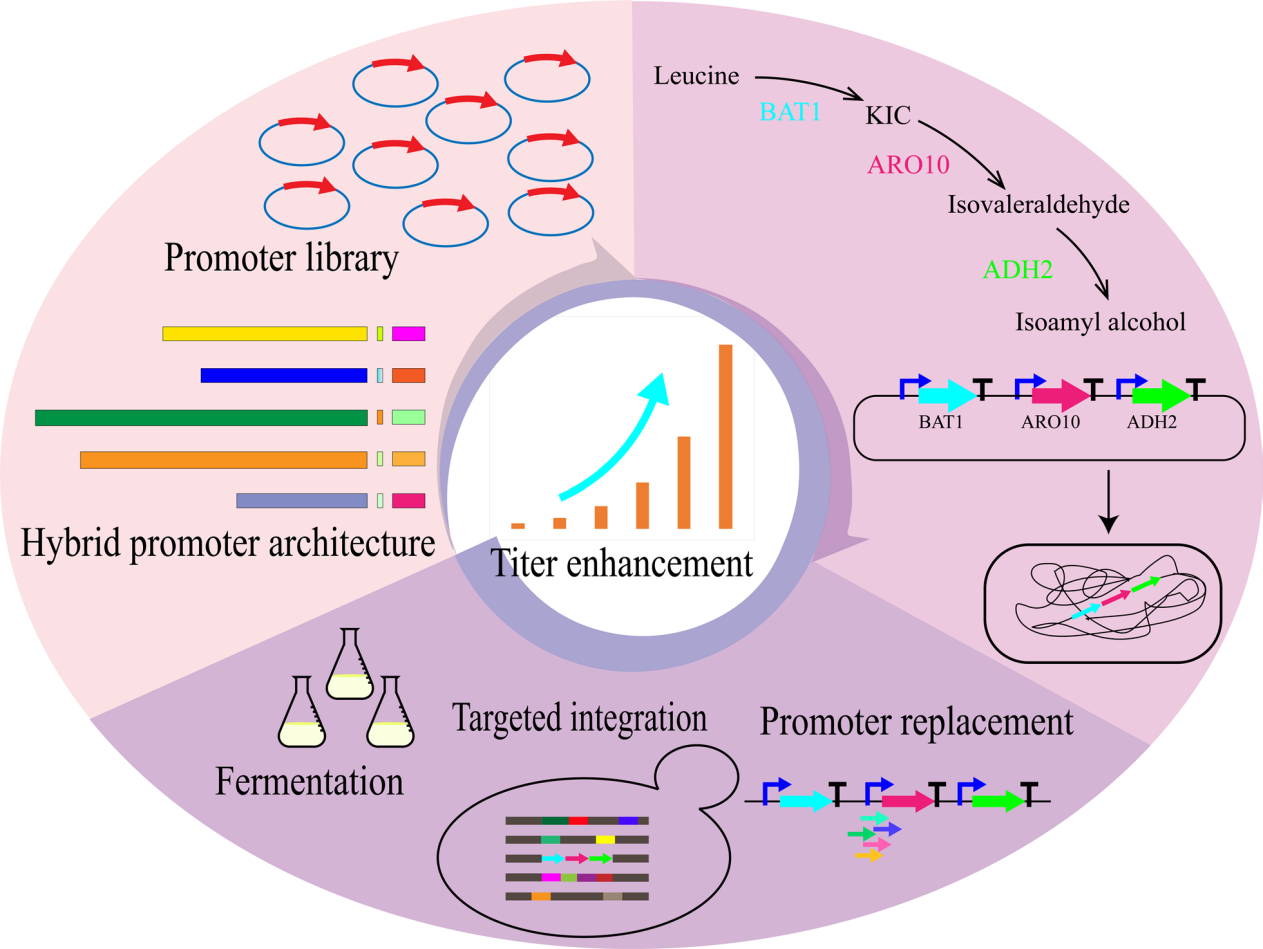
Strategy of promoter engineering using isoamyl alcohol production as the test study. An artificial hybrid promoter library that covers a wide range of promoter strength was constructed, and applied to optimize the isoamyl alcohol synthesis pathway in *Y. lipolytica*

In this study, to explore the mechanism of synergy between various elements in *Y. lipolytica*, the promoter elements were characterized and rearranged. Consequently, a library of hybrid promoters that enables stable expression and covers a wide range of promoter strength was constructed. Subsequently, we employed the hybrid promoters for promoter engineering of the pathway genes in isoamyl alcohol biosynthesis. Isoamyl alcohol, an important platform chemical, is widely applied in the production of biofuels, fragrances, medicines and fine chemicals [13] and has been produced in recent years by metabolic engineering of diverse microbial cells, such as *Escherichia coli*, *Corynebacterium glutamicum* and *Aspergillus oryzae* [14–16]. While there is a report on improving the production of isoamyl alcohol in *Y. lipolytica* by metabolic engineering, a native promoter was employed [17]. Therefore, we used this pathway as a testbed and demonstrated the efficacy of our promoter library for optimizing metabolic pathways by significantly improving the isoamyl alcohol titer (Fig. 1). The outcome of this work shows that promoter engineering is an effective strategy for facilitating metabolic engineering efforts to biosynthesize valuable chemicals and our hybrid promoter library is a powerful toolkit for future metabolic engineering work in *Y. lipolytica*.

## Results and discussion

### Characterization of native promoters as a basis for the construction of hybrid promoters for *Yarrowia lipolytica*

The strengths of different native promoters are known to vary greatly in microbes. We systematically characterized the expression of different GFPs to determine the gene that may function as an ideal reporter in *Y. lipolytica* Po1g *KU70*∆, which was the host strain for this work. The results show that *hrGFPO* was the most suitable reporter gene, as its fluorescence was high and consistent. Thus, *hrGFPO* was utilized for subsequent promoter characterization (Additional file 1: Figures S1, S2).

**Fig. 2:**
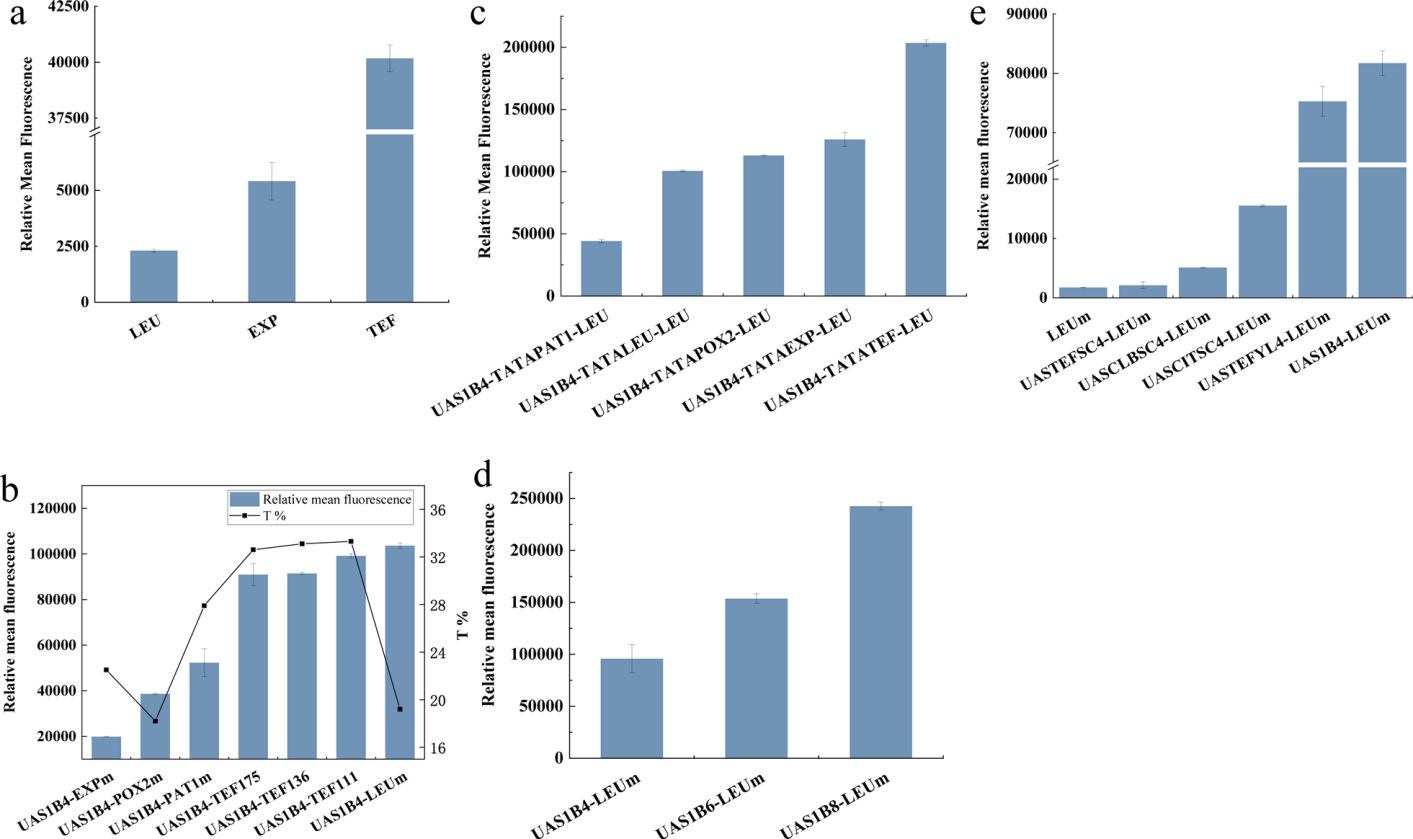
Fluorescence strength of the promoters. **a** Characterization of the native promoters P_*LEU*_, P_*EXP*_ and P_*TEF*_. **b** Characterization of different core promoters, and the relationship between T content upstream of TSS and relative mean fluorescence. Bars represent relative mean fluorescence and lines represent percentage of T content upstream of TSS. **c** Characterization of different TATA boxes. **d** Relationship between the copy number of UAS and relative mean fluorescence. **e** Characterization of different UASs from *S. cerevisiae* and *Y. lipolytica*. At least three biological replicates were measured by flow cytometry, and a cell count of 10,000 was analyzed. The error bars represent standard deviation

**Fig. 3:**
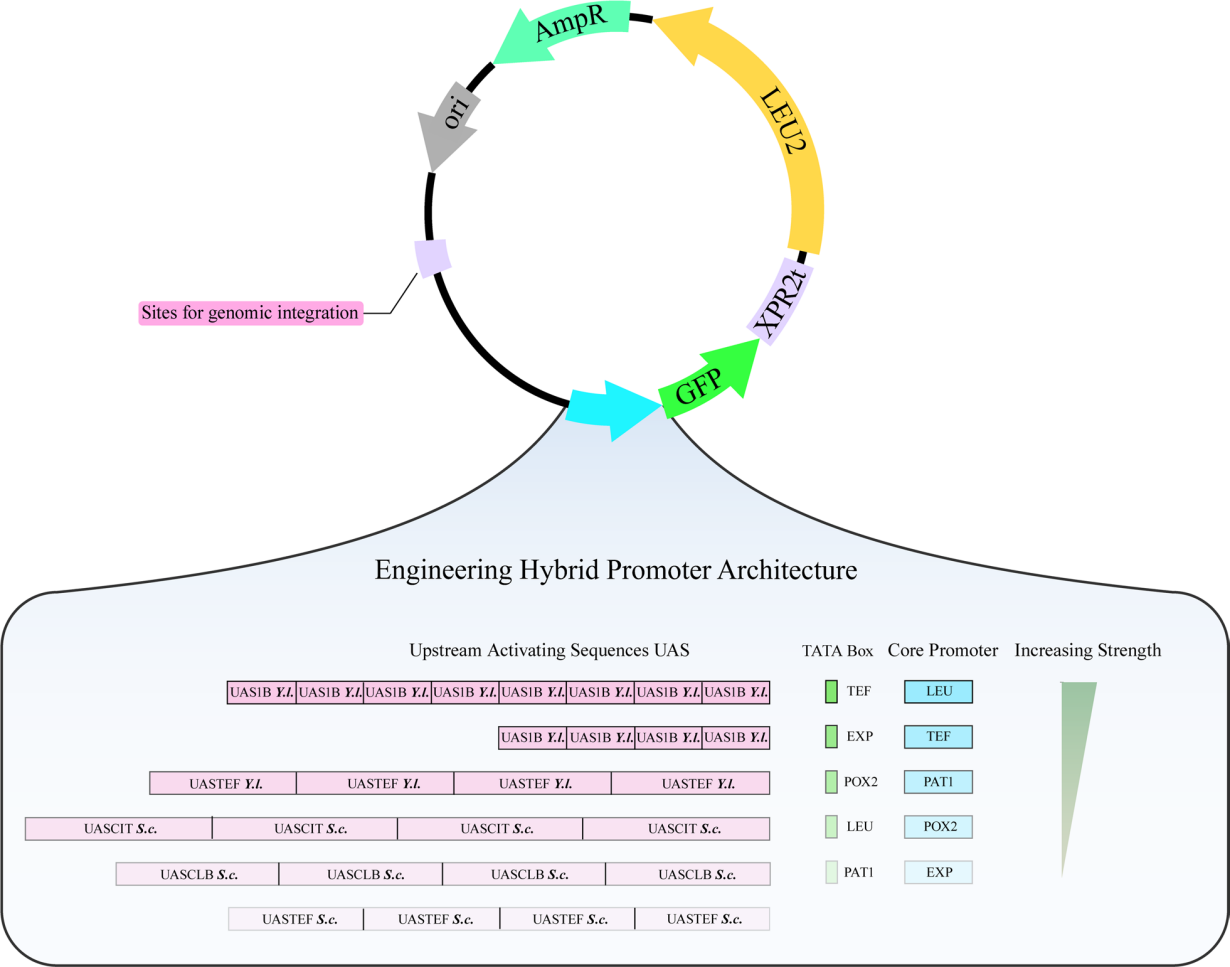
Strategy of engineering hybrid promoter architecture. Different promoter elements (UAS, TATA box and core promoter) were tested and ligated to the upstream of the reporter gene *hrGFPO* to characterize promoter strength in this study

To form a basis for our hybrid promoter library, we sought to use the *hrGFPO* reporter gene to evaluate the promoter strengths of several commonly used native *Y. lipolytica* promoters: β-isopropylmalate dehydrogenase (*LEU2*) gene promoter P_*LEU*_, export protein (*EXP1*) gene promoter P_*EXP*_ and translation elongation factor-1α (*TEF1*) gene promoter P_*TEF*_. Based on the results of our experiments (Fig. 2a), the relative fluorescence intensities of the corresponding strains from high to low are P_*TEF*_ > P_*EXP*_ > P_*LEU*_, whereby the strength of P_*TEF*_ is about an order stronger than both P_*EXP*_ and P_*LEU*_. Subsequently, these promoters were dissected into the various promoter elements, i.e., UAS, TATA box and core promoter. Based on the structures of these native promoters, other known promoter elements were added to build hybrid promoters. In most previous studies on the construction on hybrid promoters, the focus was mainly on the utilization of UAS but few studies explored varying the other promoter elements. Thus, in this study, we investigated the mixing of promoter constituent elements and examined the influence of various combinations on the strengths of the resulting hybrid promoters in *Y. lipolytica* (Table 1, Fig. 3).

**Table 1:** List of promoters used in this study

Promoters	UAS type	TATA box	Core promoter	Strength	References
LEU				+	[40]
EXP				+	[31]
TEF				+ +	[31]
LEUm		LEU	LEU	+	[12]
UASTEFSC4-LEUm	UASTEFSC4	LEU	LEU	+	This study
UASCLBSC4-LEUm	UASCLBSC4	LEU	LEU	+	This study
UASCITSC4-LEUm	UASCITSC4	LEU	LEU	+ +	This study
UAS1B4-EXPm	UAS1B4	EXP	EXP	+ +	This study
UAS1B4-POX2m	UAS1B4	POX2	POX2	+ +	This study
UAS1B4-TATAPAT1-LEU	UAS1B4	PAT1	LEU	+ +	This study
UAS1B4-PAT1m	UAS1B4	PAT1	PAT1	+ + +	This study
UASTEFYL4-LEUm	UASTEFYL4	LEU	LEU	+ + +	This study
UAS1B4-TEF175	UAS1B4	TEF	TEF175	+ + +	This study
UAS1B4-TEF136	UAS1B4	TEF	TEF136	+ + +	This study
UAS1B4-TEF111	UAS1B4	TEF	TEF111	+ + +	This study
UAS1B4-LEUm	UAS1B4	LEU	LEU	+ + + +	[20]
UAS1B4-TATAPOX2-LEU	UAS1B4	POX2	LEU	+ + + +	This study
UAS1B4-TATAEXP-LEU	UAS1B4	EXP	LEU	+ + + +	This study
UAS1B6-LEUm	UAS1B6	LEU	LEU	+ + + + +	This study
UAS1B4-TATATEF-LEU	UAS1B4	TEF	LEU	+ + + + +	This study
UAS1B8-LEUm	UAS1B8	LEU	LEU	+ + + + +	[26]

+ Means the range of relative mean fluorescence is 0–10,000; + + means the range of relative mean fluorescence is 10,000–50,000; + + + means the range of relative mean fluorescence is 50,000–100,000; + + + + means the range of relative mean fluorescence is 100000–150,000; + + + + + means the range of relative mean fluorescence is > 150,000

### Characterization of features in core promoters that influence promoter strength

The core promoter, first identified in the mammalian gene regulatory region, plays a key role in regulating the initiation of gene transcription and is defined as ‘the smallest DNA element for transcription’ [3]. In yeast systems, many studies have proved that the regulation mechanism of the core promoter has a very complex impact on the activity and strength of the promoter, and thus modulate gene expression. For example, in *S. cerevisiae*, the T content in the core promoter upstream of the TSS has a pronounced influence on the promoter activity. When the gene expression was high, the T content upstream of the TSS was abundant, and the A content downstream of TSS was rich [18]. Thus, we hypothesized that a similar trend exists in *Y. lipolytica*. Therefore, a series of endogenous core promoters of different lengths and which contain a TATA box, namely, LEU, TEF, EXP, POX2 and PAT1, were selected to calculate the content of T upstream of the TSS and verify the functions of the core promoters in *Y. lipolytica*. To confirm the function of the core promoters, the UAS1B4 elements which activate gene transcription were linked to the upstream of the core promoter to express the *hrGFPO* reporter gene for characterizing the promoter strengths by fluorescence. The results show that the promoter strengths of the hybrid promoters that we constructed in general increased with the T content upstream of the TSS (Fig. 2b), implying that the activity of the promoter was related to the upstream T content of the TSS [1, 18]. Nevertheless, two exceptions, LEUm and POX2m, suggest that other factors may be involved in determining the promoters’ activity, e.g., co-regulation by known elements such as UAS and TATA box studied in this study.

We further investigated the effect of promoter truncation on the activity of the TEF core promoter by analyzing the promoters TEF111, TEF136 [1] and TEF175, which are truncated P_*TEF*_ from the 3′ terminal with lengths of 111, 136 and 175 bp, respectively (Additional file 1: Figure S3). The results indicate that the hybrid promoter strength increases with decreasing length of the core promoter in the promoter P_*TEF*_ for *Y. lipolytica* (Fig. 2b). These data suggest that expression level of genes can be regulated largely by both the types and length of core promoters. While there appears to be a relationship between T content and promoter strength in *Y. lipolytica*, further studies are required to elucidate the specific relationship between the base content and promoter strength.

### Modulating the promoter strength by varying the TATA box

Functional elements of the core promoter including TATA box, initiator element, downstream promoter element, TFIIB recognition element and motif ten element have been identified [19, 20]. The sequence lengths of these functional elements are short, the specificities are low and the combinations in various promoters are different. All these functional elements, except the TATA box, are clearly nonconserved in yeast [21, 22]. The TATA box, which is the binding site of the TATA binding protein (TBP), is the first element identified in the core promoter. Previous studies have shown that the TATA box has a significant effect on promoter strength [1]. Therefore, a series of TATA boxes (Table 2) were selected to study their specific performance in promoters in *Y. lipolytica*. P_*UAS1B4*+*LEU*_, which has the highest activity among the hybrid promoters constructed in the previous section, was selected as the control for engineering. First, we selected several TATA boxes to replace TATA LEU by site-directed mutagenesis. The expression of *hrGFPO* under the promoter variants was evaluated by fluorescence and it was shown that different TATA boxes significantly affected the promoter strength (Fig. 2c). Notably, the fluorescence intensity of the strain with the hybrid promoter containing TATA TEF was more than twice that of the control strain with TATA LEU. Therefore, the results validate the important role of TATA box in influencing the strength of a promoter and provide a theoretical basis for future promoter engineering studies.

**Table 2:** TATA box tested in this study

TATA box	Sequence
LEU	TATATATA
TEF	TATAAAA
EXP	ATTATATATAA
PAT1	TATATACC
POX2	GTATACTTATATA

### Construction of promoters with various UAS elements from *Yarrowia lipolytica* and *Saccharomyces cerevisiae*

The process of transcriptional regulation begins with the recognition of specific sequences by transcription factors (TFs), such as the recognition of UASs by transcriptional activators and upstream repression sequences (URSs) by repressors. Many studies have shown that UAS has a strong influence on transcriptional regulation. Several UASs have been identified in *S. cerevisiae*, such as UASTEF [23], UASCLB [24] and UASCIT [25]. However, only a few UASs have been identified in *Y. lipolytica*, among which the UAS1B is the most well-studied. In previous studies, it has been demonstrated that the copy number of UAS has a significant impact on hybrid promoter strength as well [2, 12]. Four tandem UAS1B of P_*XPR2*_ and one P_*LEU*_ core promoter have been combined to construct the strong constitutive promoter P_*UAS1B4*+*LEUm*_ [26]. We increased the copy number of UAS and verified that the copy number of UAS is proportional to the hybrid promoter strength (Fig. 2d), which corroborates with published data [26]. In addition, while it has been shown that synthetic terminators can be effectively transferred between *S. cerevisiae* and *Y. lipolytica* [27], there is no research on the transferability of promoter elements across diverse yeast species. Therefore, different UASs (UASCIT *S.c*., UASCLB *S.c.*, UASTEF *S.c.* and UASTEF *Y.l.*) [2, 23–25] from *S. cerevisiae* and *Y. lipolytica* were used to replace UAS1B4 in P_*UAS1B4*+*LEUm*_ at the same copy number to explore the influence of UAS types and origin on hybrid promoter activity. By expressing the *hrGFPO* gene under the hybrid promoters with different UASs, the activities of the promoters were proved to be significantly affected by the variation in UAS. The relative fluorescence intensity from the GFP expressed from the promoters containing various UASs, from high to low, is UAS1B > UASTEF *Y.l.* > UASCIT *S.c.* > UASCLB *S.c.* > UASTEF *S.c.* (Fig. 2e). These results demonstrate for the first time that UAS from *S. cerevisiae* are functional in *Y. lipolytica*.

**Fig. 4:**
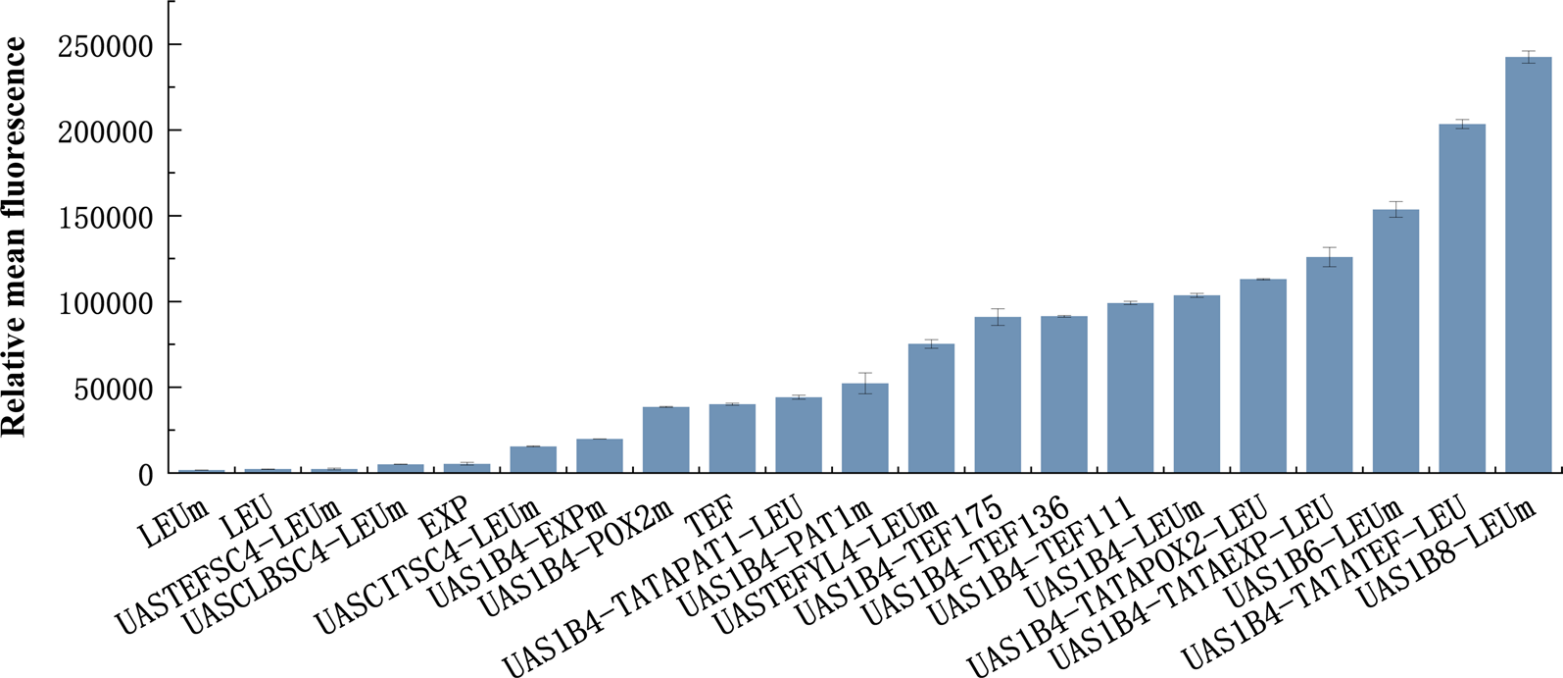
Fluorescence of the promoter library constructed in this study. The *hrGFPO* was used as a reporter gene for the promoter library constructed, and the fluorescence were detected by the BD Accuri C6 flow cytometer (BD Biosciences) using 488-nm excitation wavelength and FL1 channel. The error bars represent standard deviation

Taken together, we have constructed a library of hybrid promoters with different promoter strengths using various combinations of UASs, TATA boxes and core promoters, as summarized in Fig. 4 and Table 1. As a testbed to demonstrate the application of our hybrid promoter library, we aimed to optimize a biosynthesis pathway, i.e., isoamyl alcohol production, by promoter engineering using our hybrid promoters to regulate gene expression and improve the production level of the target compound.

### Construction of the isoamyl alcohol overexpression pathway in *Yarrowia lipolytica*

**Fig. 5:**
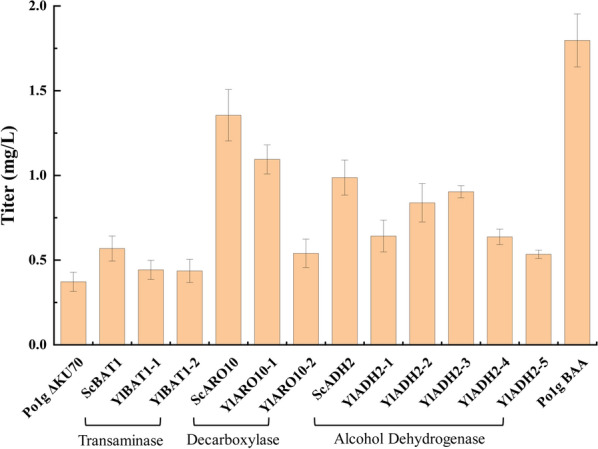
Production of isoamyl alcohol in engineered *Y. lipolytica*. The cultures were grown in 40 mL YPD medium with an initial OD_600_ of 0.1 and 10% of *n*-dodecane in a 250 mL shake flask at 225 rpm and 28 °C for 3 days. The organic phase was analyzed by GC/MS. *ScARO10* is the key gene in the heterologous isoamyl alcohol production pathway. The isoamyl alcohol titer of Po1g ScARO10, which expressed *ScARO10* under P_*UAS1B4-LEUm*_, was 1.36 mg/L, which was 2.7-fold higher than the control strain Po1g *KU70*Δ. The strain Po1g BAA, which co-expressed *ScBAT1*, *ScARO10* and *ScADH2* under P_*UAS1B4-LEUm*_, achieved an isoamyl alcohol titer of 1.8 mg/L, which was 3.9-fold that of control strain Po1g *KU70*Δ. The error bars represent standard deviation

As an important platform chemical, isoamyl alcohol is a promising biofuel and biochemical with a huge market demand. However, the titer of isoamyl alcohol in *Y. lipolytica* is quite low natively at a mere 0.37 mg/L (Fig. 5). Thus, the production titer of isoamyl alcohol has much room for improvement and the biosynthesis pathway serves as a suitable testbed for optimization by promoter engineering using our hybrid promoter library.

In yeast, isoamyl alcohol is generally produced through the Ehrlich pathway, which usually involves three reaction steps: transamination, decarboxylation and reduction. Twelve genes encoding transaminases (*ScBAT1*, *YlBAT1-1* and *YlBAT1-2*), decarboxylases (*ScARO10*, *YlARO10-1* and *YlARO10-2*) and alcohol dehydrogenases (*ScADH2*, *YlADH2-1*, *YlADH2-2*, *YlADH2-3*, *YlADH2-4* and *YlADH2-5*) were selected and individually overexpressed to determine the key genes of isoamyl alcohol biosynthesis in the Ehrlich pathway. For this purpose, twelve strains overexpressing native and heterologous genes in the Ehrlich pathway were constructed. All genes were individually integrated into the genome of *Y. lipolytica* Po1g *KU70*Δ and driven by the constitutive promoter P_*UAS1B4*+*LEUm*_. After 3 days of cultivation, individual overexpression of the pathway genes enhanced the isoamyl alcohol titer in the engineered strains compared to that of the control strain Po1g *KU70*Δ (Fig. 5). The results show that among the three evaluated classes of enzymes in the Ehrlich pathway, the strains overexpressing decarboxylase genes resulted in the most significant increase in isoamyl alcohol production. Among them, the highest isoamyl alcohol production was obtained by the *ScARO10*-overexpressed strain, which reached 1.36 mg/L. The strains that overexpressed the transaminase gene *ScBAT1* and the dehydrogenase gene *ScADH2* also increased the production of isoamyl alcohol moderately. The results indicate that the decarboxylase encoded by *ScARO10* is the key limiting step in the Ehrlich pathway, which improved isoamyl alcohol biosynthesis in *Y. lipolytica* upon overexpression (Fig. 5). To further improve the yield of isoamyl alcohol, the genes *ScBAT1*, *ScARO10* and *ScADH2* were chosen for overexpression to construct the strain Po1g BAA. After 3 days of cultivation, the titer of isoamyl alcohol reached 1.8 mg/L, which was 3.9-fold higher than that of the control strain Po1g *KU70*Δ (Fig. 5). Thus, strain Po1g BAA was selected for subsequent engineering by promoter replacement with our hybrid promoter library.

### Application of the hybrid promoter library to improve the isoamyl alcohol biosynthesis pathway

**Fig. 6:**
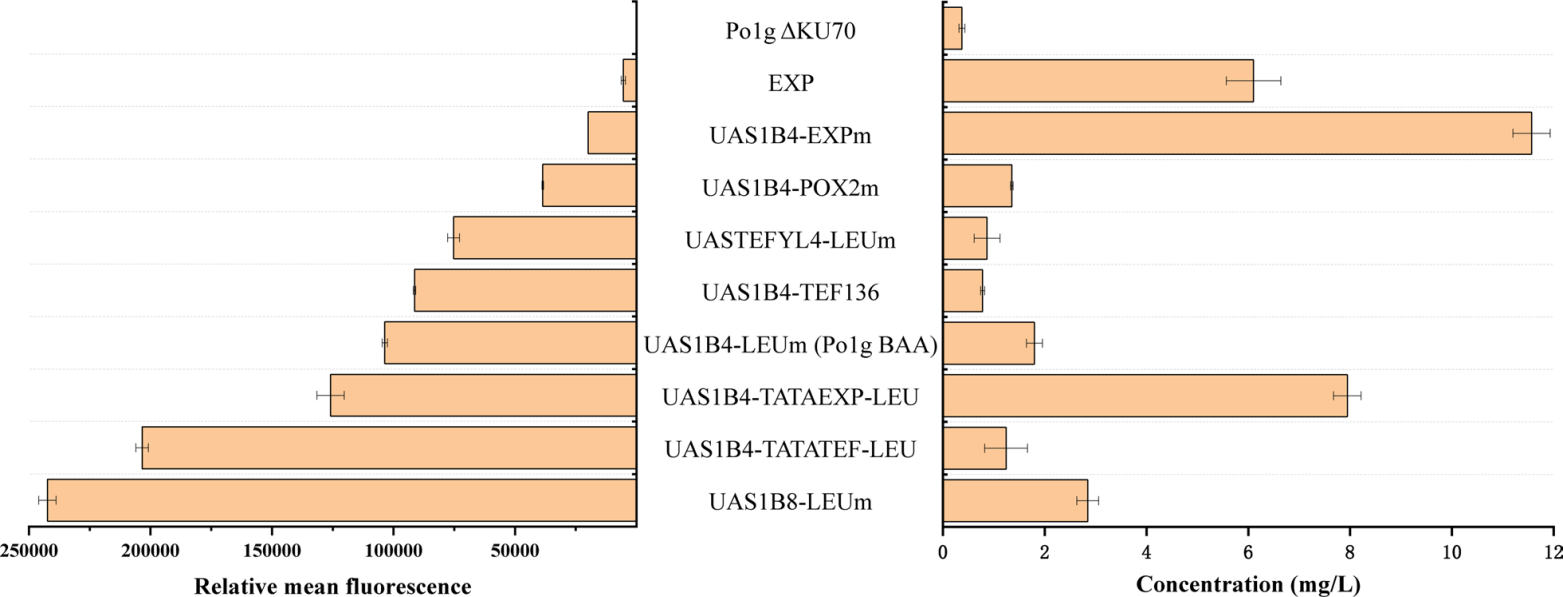
Comparison of partial promoter strength with isoamyl alcohol titer. Several promoters that cover a range of strengths (left) were selected to replace P_*UAS1B4-LEUm*_ for overexpressing *ScARO10* and the titers of isomayl alcohol were measured (right). The strain with P_*UAS1B4-EXPm*_ achieved the highest isoamyl alcohol titer of 11.57 mg/L, which was approximately 30.3-fold higher than that of Po1g *KU70*Δ and 5.4-fold that of Po1g BAA. The error bars represent standard deviation

In metabolic engineering, studies have shown that the yield of the target product can be increased by replacing promoters of pathway genes with stronger ones [28, 29]. Therefore, to demonstrate the application of our promoter library for optimizing metabolic pathways, we employed our hybrid promoters in the heterologous isoamyl alcohol pathway of Po1g BAA. Nine representative promoters were selected from our hybrid promoter library to cover a wide spectrum of strengths for optimizing the expression of *ScARO10*, a gene that can overcome a major bottleneck in the isoamyl alcohol pathway when overexpressed. These constructed strains were cultured for 3 days, and the titer of the isoamyl alcohol was quantified (Fig. 6). It can be seen from the results that the isoamyl alcohol titers do not correlate with the strengths of the promoters used. For example, strain Po1g BA + P_*UAS1B4*+*EXPm*_ + ARO10 with a low-activity promoter had the highest isoamyl alcohol titer of 11.57 mg/L, which was about 30.3-fold higher than that of Po1g *KU70*Δ and 5.4-fold that of Po1g BAA. This result is consistent with the opinion of Dulermo et al*.* that stronger promoters do not necessarily increase the expression level and/or function of a protein [30]. In addition, we found that although the activity of P_*EXP*_ was low, several strains containing P_*EXP*_ elements (P_*EXP*_, P_*UAS1B4-EXPm*_, P_*UAS1B4*+*TATAEXP-LEUm*_) had higher titers of isoamyl alcohol, suggesting that the elements of the P_*EXP*_ have greater beneficial effects to the expression of the *ARO10* gene. It is unclear why the expression of *ARO10* benefitted from the elements of P_*EXP*_, which drives the expression of a necessary gene YALI0C12034g that is highly homologous to an *S. cerevisiae* encoding a non-classical export protein 2 [31]. It is possible that transcriptional/post-transcriptional regulation or protein stability and modification plays a regulatory role in the activity of decarboxylases (*ARO10*) but more studies are needed to better understand the mechanism between the P_*EXP*_ and gene expression which resulted in the improved production titer. Nevertheless, our results indicate that different promoters can affect the titer of isomayl alcohol and demonstrate the capability of using our hybrid promoter library for pathway optimization. Attempts to further improve isoamyl alcohol titer may be achieved by applying the hybrid promoter library to other key genes in the pathway. Further understanding of the relationship between promoter elements, genes and gene expression will offer valuable insights to facilitate high production of the target compounds.

## Conclusions

Promoters are one of the most essential components of synthetic biology for determining protein expression. Compared to prokaryotes, the regulatory mechanism of the promoter structure in eukaryotes is extremely complex [1, 32]. Increasing the promoter strength is a common method to improve gene transcription and protein expression level. However, recent studies have revealed that not all strong promoters can achieve the highest protein expression and activity [30]. We explored the structure and functional characteristics of the promoters in *Y. lipolytica*, and subsequently constructed a series of promoters which are stable and efficient in this study. This is the first time that the T content upstream of the TSS has been shown to positively correlate with the hybrid promoter strength in *Y. lipolytica*, although it is worth noting that some core promoter elements, such as POX2m and LEUm, did not conform to the trend. Therefore, the relationship between the T content upstream of the TSS and the promoter strength in *Y. lipolytica* needs to be further studied. Next, the effects of different UAS elements from *S. cerevisiae* and *Y. lipolytica* on promoter strength were investigated and we discovered for the first time that UAS elements can be transferred between yeast species. However, the functions of the UASs from *S. cerevisiae* were weaker than the native ones from *Y. lipolytica* (Fig. 2e), and the underlying molecular mechanisms need to be investigated further in future work. Nevertheless, these findings lay the groundwork for the development of hybrid promoters which can be effectively transferred across diverse yeast species.

To demonstrate application of our hybrid promoter library, the isoamyl alcohol production pathway was constructed to serve as a testbed by co-expressing multiple genes from *S. cerevisiae* and *Y. lipolytica*. *ScARO10*, the key gene of the isoamyl alcohol pathway, was selected as the test gene for expression under various hybrid promoters from our library to optimize the enzyme’s expression and activity for enhancing isoamyl alcohol production. Consequently, the titer of the isoamyl alcohol was 30.3-fold higher than the control strain. Although the titer of isoamyl alcohol from *Y. lipolytica* achieved here is lower compared with other studies [17, 33, 34], it is the first time that promoter engineering has been applied for the biosynthesis of isoamyl alcohol to provide an advanced solution for the production of biofuels and alcohols. Regulation of expression by promoters involves numerous factors, such as temperature, pH and substrate [1, 35]. In the future, we will further study the mechanisms of promoters to construct hybrid promoters with stronger activity and a wider range of expression levels for optimum expression of biosynthesis pathway genes to achieve high-level production of value-added chemicals.

## Materials and methods

### Strains and media

*Escherichia coli* strain DH5α was used for all cloning and plasmid propagation, and DH5α was grown at 37 °C in Luria Bertani (LB), and supplemented with ampicillin to final concentration of 100 μg/mL for plasmid propagation. *Yarrowia* *lipolytica* strain Po1g *KU70*Δ, a leucine auxotroph devoid of any secreted protease activity, was used as the base strain in this study. The original plasmid pYLEX1, a pBR322-based monocopy integrative vector [12, 36], is produced by Yeastern Biotech Co., Ltd. *Y. lipolytica* Po1g *KU70*Δ was fitted with an integrated pBR322 docking platform. The vector pYLEX1 digested with *Spe* I or *Not* I will be inserted to the pBR322 locus of the Po1g *KU70*Δ strain. After transformation, the positive *Y. lipolytica KU70* Δ transformants were selected on YNB-leu plates and subsequently confirmed by genomic DNA PCR analysis. *Yarrowia lipolytica* Po1g *KU70* Δ containing plasmid was routinely cultivated at 28 °C and 225 rpm with YPD media consisting of 20 g/L glucose, 20 g/L peptone, and 10 g/L yeast extract. In this study, PCR primers were synthesized by Genewiz (Jiangsu, China) and are listed in Additional file 1: Table S1, plasmids are listed in Additional file 1: Table S2 and strains used are listed in Additional file 1: Table S3.

### Chemicals and enzymes

All restriction enzymes were purchased from New England Biolabs (Beijing, China), 2× Phanta^®^ max master mix, 2× Rapid Taq master mix, ClonExpress^®^ II one step cloning kit, FastPure^®^ Plasmid Mini Kit and FastPure^®^ Gel DNA Extraction Mini Kit were purchased from Vazyme Biotech Co., Ltd. (Nanjing, China), peptone and yeast extract were purchased from Thermo Scientific Oxoid Microbiology Products (Basingstoke, England), isoamyl alcohol and n-dodecane were purchased from Aladdin^®^ (Shanghai, China).

### Plasmid construction of promoter library

The *GFPuv* gene was preserved in this laboratory, and cloned into pYLEX1 with primers GFPuv-F/GFPuv-R (Additional file 1: Table S1) yield plasmid pYLGFPuv (Additional file 1: Table S2). The *hrGFP* gene and *hrGFPO* gene were synthesized and cloned into pYLEX1 to yield plasmids pYLhrGFP and pYLhrGFPO (Additional file 1: Table S2), respectively, by Genewiz (Jiangsu, China). The UASCIT *S.c.*4, UASCLB *S.c.*4, UASTEF *S.c.*4, UASTEF *Y.l.*4, UAS1B6 and UAS1B8 motifs were synthesized and cloned into plasmids pYLhrGFPO to replace UAS1B4 to yield plasmids pYLP_*UASCITSC4-LEUm*_ + hrGFPO, pYLP_*UASCLBSC4-LEUm*_ + hrGFPO, pYLP_*UASTEFSC4-LEUm*_ + hrGFPO, pYLP_*UASTEFYL4-LEUm*_ + hrGFPO, pYLP_*UAS1B6-LEUm*_ + hrGFPO and pYLP_*UAS1B8-LEUm*_ + hrGFPO (Additional file 1: Table S2), respectively, by Genewiz (Jiangsu, China). Three endogenous promoters P_*LEU*_, P_*TEF*_ and P_*EXP*_ were cloned into vector pYLhrGFPO with primers PLEU-F/LEU-hrGFPO-R, PTEF-F/TEF-hrGFPO-R and PEXP-F/EXP-hrGFPO-R (Additional file 1: Table S1) yield plasmids pYLP_*LEU*_ + hrGFPO, pYLP_*TEF*_ + hrGFPO and pYLP_*EXP*_ + hrGFPO (Additional file 1: Table S2), respectively. The core promoters were amplified by primer pairs PAT1m-F/PAT1-hrGFPO-R, POX2m-F/POX2-hrGFPO-R, EXPm-F/EXP-hrGFPO-R, TEFm111-F/TEF-hrGFPO-R, TEFm136-F/TEF-hrGFPO-R and TEFm175-F/TEF-hrGFPO-R (Additional file 1: Table S1), and then replace the core promoter LEU in P_*UAS1B4-LEU*_. These promoters were ligated to pYLhrGFPO in place of the P_*UAS1B4-LEU*_ to yield plasmids pYLP_*UAS1B4-PAT1m*_ + hrGFPO, pYLP_*UAS1B4-POX2m*_ + hrGFPO, pYLP_*UAS1B4-EXP1m*_ + hrGFPO, pYLP_*UAS1B4-TEF111*_ + hrGFPO, pYLP_*UAS1B4-TEF136*_ + hrGFPO and pYLP_*UAS1B4-TEF175*_ + hrGFPO (Additional file 1: Table S2), respectively. The TATA box LEU in P_*UAS1B4-LEU*_ was replaced by TATA box TEF, EXP, PAT1 and POX2 using primer pairs TATA TEF-F/LEU-hrGFPO-R, TATA EXP-F/LEU-hrGFPO-R, TATA PAT1-F/LEU-hrGFPO-R and TATA POX2-F/LEU-hrGFPO-R (Additional file 1: Table S1). These hybrid promoters were ligated to pYLhrGFPO in place of the P_*UAS1B4-LEU*_ to yield plasmids pYLP_*UAS1B4-TATATEF-LEU*_ + hrGFPO, pYLP_*UAS1B4-TATAEXP-LEU*_ + hrGFPO, pYLP_*UAS1B4-TATAPAT1-LEU*_ + hrGFPO and pYLP_*UAS1B4-TATAPOX2-LEU*_ + hrGFPO (Additional file 1: Table S2), respectively.

All plasmids, linearized by *Not* I or *Spe* I, were transformed into competent cells of *Y. lipolytica* strains using the lithium acetate method [37].

### Plasmid construction of exogenous isoamyl alcohol pathway

The transaminase gene (*BAT1*, GenBank ID: 856615), decarboxylase gene (*ARO10*, GenBank ID: 851987) and alcohol dehydrogenase gene (*ADH2*, GenBank ID: 855349) from *S. cerevisiae* S288C were codon-optimized and synthesized and cloned into pYLEX1 to yield plasmids pYLSCBAT1, pYLSCARO10 and pYLSCADH2 (Additional file 1: Table S2), respectively, by Genewiz (Jiangsu, China). In *Y. lipolytica*, the homologous sequences that *YlBAT1-1* and *YlBAT1-2* of *ScBAT1* were cloned into pYLEX1 with primers YLBAT1-1-F/YLBAT1-1-R and YLBAT1-2-F/YLBAT1-2-R (Additional file 1: Table S1) to yield plasmids pYLYLBAT1-1 and pYLYLBAT1-2 (Additional file 1: Table S2), respectively. The homologous sequences that *YlARO10-1* and *YlARO10-2* of *ScARO10* were cloned into pYLEX1 with primers YLARO10-1-F/YLARO10-1-R and YLARO10-2-F/YLARO10-2-R (Additional file 1: Table S1) to yield plasmids pYLYLARO10-1 and pYLYLARO10-2 (Additional file 1: Table S2), respectively. The homologous sequences that *YlADH2-1*, *YlADH2-2*, *YlADH2-3*, *YlADH2-4* and *YlADH2-5* of *ScADH2* were cloned into pYLEX1 with primers YLADH2-1-F/YLADH2-1-R, YLADH2-2-F/YLADH2-2-R, YLADH2-3-F/YLADH2-3-R, YLADH2-4-F/YLADH2-4-R and YLADH2-5-F/YLADH2-5-R (Additional file 1: Table S1) to yield plasmids pYLYLADH2-1, pYLYLADH2-2, pYLYLADH2-3, pYLYLADH2-4 and pYLYLADH2-5 (Additional file 1: Table S2), respectively.

The expression cassettes of *ScARO10* and *ScADH2* were cloned into pYLSCBAT1 with primers BDH-ADH2-F/BDH-ADH2-R and BDH-ARO10-F/ BDH-ARO10-R (Additional file 1: Table S1) to yield plasmid pYLBAA (Additional file 1: Table S2). All plasmids, linearized by *Not* I or *Spe* I, were transformed into competent cells of *Y. lipolytica* strains using the lithium acetate method [37].

### Expressing the isoamyl alcohol synthesis pathway using the promoter library

Several promoters from the promoter library were used to express the *ARO10* gene which is the key gene in the isoamyl alcohol pathway. The promoters P_*EXP*_ and P_*UAS1B4*+*EXPm*_ were amplified by primers BDH-ARO10-F/PEXP-ARO10-R (Additional file 1: Table S1), and then ligated to *ScARO10* in pYLBAA to yield plasmid pYLBA + P_*EXP*_ + ARO10 and pYLBA + P_*UAS1B4-EXPm*_ + ARO10 (Additional file 1: Table S2), respectively. The promoters P_*UAS1B4-POX2m*_ and P_*UAS1B4-TEF136*_ were amplified by primers BDH-ARO10-F/POX2-ARO10-R and BDH-ARO10-F/PTEF-ARO10-R (Additional file 1: Table S1), and then ligated to *ScARO10* in pYLBAA to yield plasmid pYLBA + P_*UAS1B4-POX2m*_ + ARO10 and pYLBA + P_*UAS1B4-TEF136*_ + ARO10 (Additional file 1: Table S2), respectively. The promoters P_*UASTEFLY4-LEUm*_, P_*UAS1B4-TATAEXP-LEU*_, P_*UAS1B4-TATATEF-LEU*_ and P_*UAS1B8- LEUm*_ were amplified by primers BDH-ARO10-F/PLEU-ARO10-R (Additional file 1: Table S1), and then ligated to *ScARO10* in pYLBAA to yield plasmids pYLBA + P_*UASTEFLY4-LEUm*_ + ARO10, pYLBA + P_*UAS1B4-TATAEXP-LEU*_ + ARO10, pYLBA + P_*UAS1B4-TATATEF-LEU*_ + ARO10 and pYLBA + P_*UAS1B8-LEUm*_ + ARO10 (Additional file 1: Table S2), respectively.

All plasmids, linearized by *Not* I or *Spe* I, were transformed into competent cells of *Y. lipolytica* strains using the lithium acetate method [38].

### Yeast strain construction

The competent cell scheme and transformation method are referred to Pang et al.[39]. After selection, the following engineered *Y. lipolytica* strains were generated: Po1g P_*UAS1B4-LEUm*_ + GFPuv, Po1g P_*UAS1B4-LEUm*_ + hrGFP, Po1g P_*UAS1B4-LEUm*_ + hrGFPO, Po1g P_*UAS1B6-LEUm*_ + hrGFPO, Po1g P_*UAS1B8-LEUm*_ + hrGFPO, Po1g P_*LEUm*_ + hrGFPO, Po1g P_*LEU*_ + hrGFPO, Po1g P_*EXP*_ + hrGFPO, Po1g P_*TEF*_ + hrGFPO, Po1g P_*UAS1B4-EXPm*_ + hrGFPO, Po1g P_*UAS1B4-POX2m*_ + hrGFPO, Po1g P_*UAS1B4-PAT1m*_ + hrGFPO, Po1g P_*UAS1B4-TEF111*_ + hrGFPO, Po1g P_*UAS1B4-TEF136*_ + hrGFPO, Po1g P_*UAS1B4-TEF175*_ + hrGFPO, Po1g P_*UAS1B4-TATAPAT1-LEUm*_ + hrGFPO, Po1g P_*UAS1B4-TATAPOX2-LEUm*_ + hrGFPO, Po1g P_*UAS1B4-TATAEXP-LEUm*_ + hrGFPO, Po1g P_*UAS1B4-TATATEF-LEUm*_ + hrGFPO, Po1g P_*UASTEFSC4-LEUm*_ + hrGFPO, Po1g P_*UASCITSC4-LEUm*_ + hrGFPO, Po1g P_*UASCLBSC4-LEUm*_ + hrGFPO, Po1g P_*UASTEFYL4-LEUm*_ + hrGFPO, Po1g ScBAT1, Po1g YlBAT1-1, Po1g YlBAT1-2, Po1g ScARO10, Po1g YlARO10-1, Po1g YlARO10-2, Po1g ScADH2, Po1g YlADH2-1, Po1g YlADH2-2, Po1g YlADH2-3, Po1g YlADH2-4, Po1g YlADH2-5, Po1g BAA, Po1g BA + P_*EXP*_ + ARO10, Po1g BA + P_*UAS1B4-EXPm*_ + ARO10, Po1g BA + P_*UAS1B4-POX2m*_ + ARO10, Po1g BA + P_*UASTEFYL4-LEUm*_ + ARO10, Po1g BA + P_*UAS1B4-TEF136*_ + ARO10, Po1g BA + P_*UAS1B4-LEUm*_ + ARO10, Po1g BA + P_*UAS1B4-TATAEXP-LEU*_ + ARO10, Po1g BA + P_*UAS1B4-TATATEF-LEU*_ + ARO10, Po1g BA + P_*UAS1B8-LEUm*_ + ARO10 (Additional file 1: Table S3).

The *Y. lipolytica* strains Po1g P_*UAS1B4-LEUm*_ + GFPuv, Po1g P_*UAS1B4-LEUm*_ + hrGFP, Po1g P_*UAS1B4-LEUm*_ + hrGFPO, Po1g P_*UASTEFYL4-LEUm*_ + hrGFPO, Po1g ScBAT1, Po1g YlBAT1-1, Po1g YlBAT1-2, Po1g ScARO10, Po1g YlARO10-1, Po1g YlARO10-2, Po1g ScADH2, Po1g YlADH2-1, Po1g YlADH2-2, Po1g YlADH2-3, Po1g YlADH2-4 and Po1g YlADH2-5 were verified by the primers pYL-F/pYL-R (Additional file 1: Table S1). The *Y. lipolytica* strain Po1g BAA was verified by the primers ADH2-CX-4–1/BAT1-YZ-R (Additional file 1: Table S1). The *Y. lipolytica* strains Po1g P_*UAS1B6-LEUm*_ + hrGFPO, Po1g P_*UAS1B8-LEUm*_ + hrGFPO, Po1g P_*LEUm*_ + hrGFPO, Po1g P_*LEU*_ + hrGFPO, Po1g P_*EXP*_ + hrGFPO, Po1g P_*TEF*_ + hrGFPO, Po1g P_*UAS1B4-EXPm*_ + hrGFPO, Po1g P_*UAS1B4-POX2m*_ + hrGFPO, Po1g P_*UAS1B4-PAT1m*_ + hrGFPO, Po1g P_*UAS1B4-TEF111*_ + hrGFPO, Po1g P_*UAS1B4-TEF136*_ + hrGFPO, Po1g P_*UAS1B4-TEF175*_ + hrGFPO, Po1g P_*UAS1B4-TATAPAT1-LEUm*_ + hrGFPO, Po1g P_*UAS1B4-TATAPOX2-LEUm*_ + hrGFPO, Po1g P_*UAS1B4-TATAEXP-LEUm*_ + hrGFPO, Po1g P_*UAS1B4-TATATEF-LEUm*_ + hrGFPO, Po1g P_*UASTEFSC4-LEUm*_ + hrGFPO, Po1g P_*UASCITSC4-LEUm*_ + hrGFPO and Po1g P_*UASCLBSC4-LEUm*_ + hrGFPO were verified by the primers CX-2/P-CX-R (Additional file 1: Table S1). The *Y. lipolytica* strains Po1g BA + P_*EXP*_ + ARO10, Po1g BA + P_*UAS1B4-EXPm*_ + ARO10, Po1g BA + P_*UAS1B4-POX2m*_ + ARO10, Po1g BA + P_*UASTEFYL4-LEUm*_ + ARO10, Po1g BA + P_*UAS1B4-TEF136*_ + ARO10, Po1g BA + P_*UAS1B4-LEUm*_ + ARO10, Po1g BA + P_*UAS1B4-TATAEXP-LEU*_ + ARO10, Po1g BA + P_*UAS1B4-TATATEF-LEU*_ + ARO10 and Po1g BA + P_*UAS1B8-LEUm*_ + ARO10 were verified by the primers ADH2-CX-F/A-CX-R (Additional file 1: Table S1).

### Flow cytometry

The green fluorescent proteins GFPuv, hrGFP and hrGFPO were selected as reporter proteins. At least three biological replicates were measured by flow cytometry. The colonies of transformants were selected from plates and grew in 5 mL of fresh YPD medium in tubes for 24 h. After that, the seed culture was diluted to OD_600_ 0.1 in 250 mL flasks containing 40 mL YPD medium. The cultures were cultivated with shaking at 225 rpm and 28 °C for 3 days. Before flow cytometric analysis, the cultures were centrifuged at 12,000 rpm for 1 min, and washed in 0.1 M phosphate-buffered saline (PBS), then resuspended in PBS. A cell count of 10,000 was analyzed with a BD Accuri C6 flow cytometer (BD Biosciences) using 488-nm excitation wavelength and FL1 channel for fluorescence detection. The CFlow software was used to analyze the data and compute mean fluorescence values. The background fluorescence was normalized when calculating the relative mean fluorescence.

### GC/MS analysis of isoamyl alcohol produced in the engineered *Yarrowia lipolytica* strains

The engineered *Y. lipolytica* transformants were selected from plate and prepared in 5 mL of fresh YPD medium in tube for 24 h. The seed culture solution was inoculated to 250 mL flasks containing 40 mL of YPD medium, starting from OD_600_ 0.1. The cultures were shaken at 225 rpm and 28 °C for 3 days. To extract isoamyl alcohol from the cultures, 10% *n*-dodecane was added to the cultures, and the mixture was vortexed for 3 min, then centrifuged at 7500 rpm for 5 min. The organic phase of 1ul was detected by GC/MS using an Agilent 7890B GC with an 5977B MSD equipped with a HP-5MS column (60 m × 0.25 mm × 0.25 μm, Agilent, Santa Clara, CA, USA). GC oven temperature was initially held at 60 °C for 2 min, and then ramped to 140 °C at a rate of 5 °C/min. It was then subsequently ramped at 10 °C/min to 280 °C and held for 5 min. The split ratio was 10:1. Helium was used as the carrier gas, with an inlet pressure of 13.8 psi. The injector was maintained at 280 °C and the ion source temperature was set to 230 °C. Final data analysis was achieved using MassHunter Workstation Software (Agilent, Santa Clara, CA, USA).

## Supplementary Information

**Additional file 1: Figure S1.** Screening of reporter genes. **Figure S2.** Characterization of different green fluorescent proteins expressed under the promoter P_*UAS1B4-LEUm*_. **Figure S3.** Schematic of theTEF core promoter. **Figure S4.** Time course of fluorescence by Po1g P_*UAS1B4-LEUm*_ + hrGFPO. **Table S1.** Primers used in PCR. **Table S2.** Plasmids used in this study. **Table S3.** Strains used in this study.

## Data Availability

All relevant data generated or analyzed during this study were included in this published article.

## Abbreviations

GC/MS: Gas chromatography/mass spectrometry
OD_600_: Optical density at 600 nm
LB medium: 0.5% Yeast extract, 1% tryptone and 1% NaCl
YPD medium: 1% Yeast extract, 2% peptone and 2% glucose
YNB plate: 2% Glucose, 0.67% yeast nitrogen base without amino acids and 2% agar
PCR: Polymerase chain reaction
YlBAT1-1: YALI0_D01265g
YlBAT1-2: YALI0_F19910g
YlARO10-1: YALI0_D06930g
YlARO10-2: YALI0_E07325g
YlADH2-1: YALI0_A16379g
YlADH2-2: YALI0_D25630g
YlADH2-3: YALI0_E17787g
YlADH2-4: YALI0_A15147g
YlADH2-5: YALI0_E07766g
