# Supplementary material for: Hybrid promoter engineering strategies in Yarrowia lipolytica: isoamyl alcohol production as a test study
Source: Biotechnol Biofuels. 2021 Jul 2;14:149. doi: 10.1186/s13068-021-02002-z (PMC8252286; doi:10.1186/s13068-021-02002-z)
Supplement: Supplementary file 1 — Additional file 1: Figure S1. Screening of reporter genes. Figure S2. Characterization of different green fluorescent proteins expressed under the promoter PUAS1B4-LEUm. Figure S3. Schematic of theTEF core promoter. Figure S4. Time course of fluorescence by Po1g PUAS1B4-LEUm + hrGFPO. Table S1. Primers used in PCR. Table S2. Plasmids used in this study. Table S3. Strains used in this study. [file 13068_2021_2002_MOESM1_ESM.docx]

**Additional file 1**

**Hybrid promoter engineering strategies in *Yarrowia lipolytica* using isoamyl alcohol as the test case**

Yu Zhao^a#^, Shiqi Liu^a#^, Zhihui Lu^a^, Baixiang Zhao^a^, Shuhui Wang^a^, Cuiying Zhang^a^, Dongguang Xiao^a^, Jee Loon Foo^b,c,d^, Aiqun Yu^a*^

**Screening of the fluorescent reporter gene in *Y. lipolytica***

Efficient engineering of microbial cell factories relies on optimizing the genetic construct of metabolic pathways to direct the carbon flux toward the desired product of interest. The key to achieving this goal is to eliminate metabolic bottlenecks and tune the expression of target gene precisely. To this end, we aim to construct a hybrid promoter library. For characterization of the promoters, it is critical to develop a stable, reliable and sensitive reporting system to monitor gene expression. A series of studies have evaluated the effectiveness of some reporters in the unconventional yeast *Y. lipolytica* for examining the strength of promoters, such as green fluorescent protein (GFP) and β-galactosidase [1, 2]. The promoter-GFP reporter gene assay is an established and widely used approach to evaluate promoter activity. It indirectly indicates the mRNA transcription level under the control of different promoters based on fluorescence, thus inferring the promoter activity [3, 4]. However, there are many variants of GFP and their applicability to *Y. lipolytica* needs to be assessed. Therefore, in this study, we systematically characterized the expression of different GFPs to investigate which gene may function as an ideal reporter gene in the yeast strain *Y. lipolytica* Po1g *KU70*∆ that was used as a host system for this work. Firstly, expression of different fluorescent reporter genes *GFPuv* (differs from the wild-type GFP by the amino acid replacements Val163Ala, Met153Thr, and Phe99Ser), *hrGFP* (humanized *Renilla reinformis* GFP) and *hrGFPO* (codon optimized *hrGFP* for *Y. lipolytica*) [1, 3, 5] were examined. These genes were driven by the hybrid promoter P*_UAS1B4-LEUm_*, which was constructed by Madzak *et al*. [6] and is the constitute promoter in the commonly used commercial integrative vector pYLEX1 for *Y. lipolytica*. Three biological replicates for four transformants of the GFPs were analyzed by fluorescence microscopy and flow cytometry (Fig. S1, Fig. S2). Fluorescence was not detected in the control strain Po1g *KU70*Δ while the *Y. lipolytica* strains carrying integrated *GFPuv* or *hrGFP* gene fluoresced at different intensities. The fluorescence produced in the strain carrying the GFPuv gene was relatively weak, indicating that this is not a good reporter gene for *Y. lipolytica*. In contrast, the detected fluorescence intensity of the strains that harbored the *hrGFPO* gene was more consistent than the strains that possessed the *hrGFP* gene (Fig. 3a). The difference in fluorescence consistency between *hrGFPO* and *hrGFP* may be due to inconsistent expression or premature termination caused by rare codons [7, 8]. Therefore, promoter activity may correspond better to the transcription and translation of *hrGFPO* than *hrGFP*. In addition, we performed a time course study to determine the optimal time of *hrGFPO* expression in *Y. lipolytica* Po1g P*_UAS1B4-LEUm_*+hrGFPO (Fig. S4). The results indicated that the fluorescence increased continuously from the beginning of cultivation and peaked on day 3. Subsequently, the fluorescence declined gradually. Henceforth, *hrGFPO* was selected as the reporter gene for promoter characterization in this work.


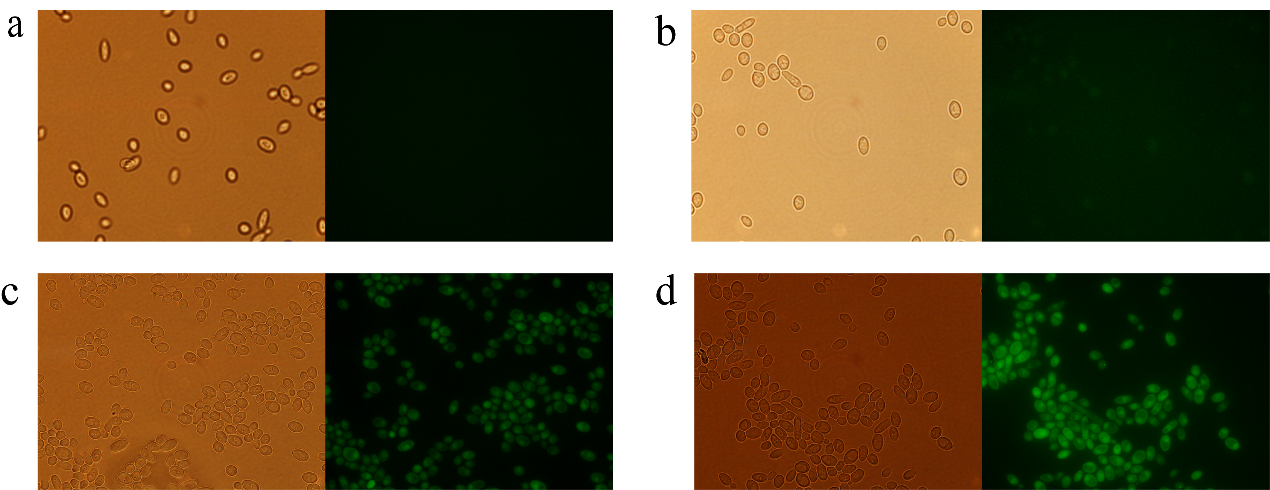


**Figure S1.** Screening of reporter genes.

a. The fluorescence image of *Y.lipolytica* Po1g *KU70*Δ with the integrative plasmid pYLEX1. b. The fluorescence image of *Y.lipolytica* Po1g *KU70*Δ with the integrative plasmid pYLGFPuv. c. The fluorescence image of *Y.lipolytica* Po1g *KU70*Δ with the integrative plasmid pYLhrGFP. d. The fluorescence image of *Y.lipolytica* Po1g *KU70*Δ with the integrative plasmid pYLhrGFPO.

**Figure S2.** Characterization of different green fluorescent proteins expressed under the promoter P*_UAS1B4-LEUm_*.

**
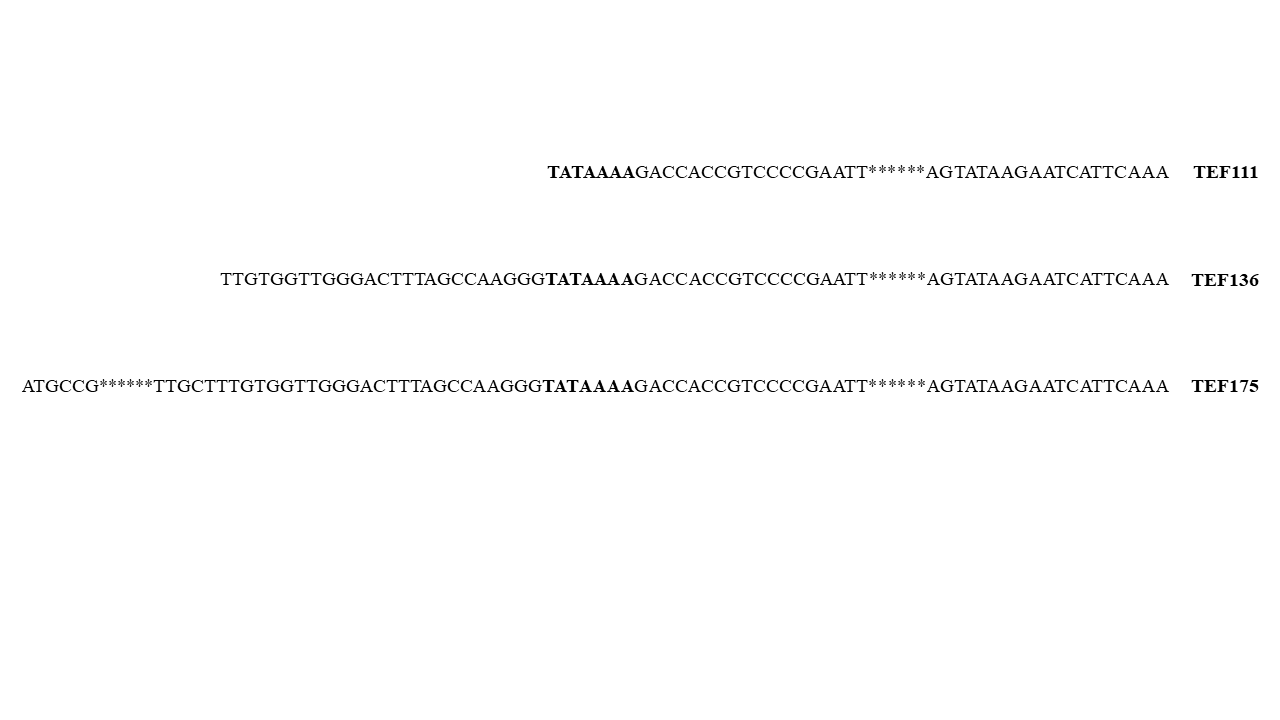
**

**Figure S3.** Schematic of TEF core promoter.





**Figure S4.** Time course of fluorescence by Po1g P*_UAS1B4-LEUm_*+hrGFPO.

**Table S1** Primers used in PCR

| Primer | Sequence (5’-3’) |
| --- | --- |
| GFPuv-F | ATACAACCACACACATCCACGTGAATGAGTAAAGGAGAAGAA |
| GFPuv-R | CCTTAGTTTCGGGTTCCCACTTATTTGTAGAGCTCATCCATGCCA |
| YLBAT1-1-F | TACAACCACACACATCCACAATGCTTCGAAACAACTTGAG |
| YLBAT1-1-R | TCCTTAGTTTCGGGTTCCCACTTAGACCACCTTAGACCAT |
| YLBAT1-2-F | TCACACATACAACCACACACATCCACAATGACCAAACTGT |
| YLBAT1-2-R | TTGGATCCTTAGTTTCGGGTTCCCACTTAGCAGTCCACGG |
| YLARO10-1-F | CAACCACACACATCCACAATGACTGCCACTTCTGCCAC |
| YLARO10-1-R | GTTTCGGGTTCCCACCTAAGGATTCTTCTTCTGGTACT |
| YLARO10-2-F | CATACAACCACACACATCCACAATGAACGGCTCGCAAGTTATCGC |
| YLARO10-2-R | CCGTAGTTGGATCCTTAGTTTCGGGTTCCCACCTACAGCTTGGAC |
| YLADH2-1-F | TACAACCACACACATCCACAATGACCACCATCCCCAAGAC |
| YLADH2-1-R | TTAGTTTCGGGTTCCCACTTACTTGGAGCAGTCCAGAACG |
| YLADH2-2-F | ACAACCACACACATCCACAATGACCACCATCCCCAAGACC |
| YLADH2-2-R | ATCCTTAGTTTCGGGTTCCCACTTACTTGTAAGTGTCCAG |
| YLADH2-3-F | CACATACAACCACACACATCCACAATGTCTGCTCCCGTCA |
| YLADH2-3-R | TGGATCCTTAGTTTCGGGTTCCCACTTACTTGGAGGTGTC |
| YLADH2-4-F | ATACAACCACACACATCCACAATGACAATCCCCAAGACCC |
| YLADH2-4-R | ATCCTTAGTTTCGGGTTCCCACTTACTTGCTGGTATCGAC |
| YLADH2-5-F | CATACAACCACACACATCCACAATGGTGAGCGACGTTCCC |
| YLADH2-5-R | GGATCCTTAGTTTCGGGTTCCCACCTACTTGCTGTTATCA |
| PLEU-F | CGCCGCAAGGAATGGTGCATGCGAATTCCGTCGTC |
| LEU-hrGFPO-R | AGGATCTGCTTAGACACCATGGTACCTGTGGATGTGTGTG |
| PTEF-F | CGCAAGGAATGGTGCATGCAGAGACCGGGTTGGCGGCGTA |
| TEF-hrGFPO-R | AGGATCTGCTTAGACACCATGGTACCTTTGAATGATTCTTA |
| PEXP-F | GCCGCAAGGAATGGTGCGAGTTTGGCGCCCGTT |
| EXP-hrGFPO-R | TCTGCTTAGACACCATGGTACCTGCTGTAGATAT |
| PAT1m-F | GCATGCTCTAGATATATACCTCGGTC |
| PAT1-hrGFPO-R | AGGATCTGCTTAGACACCATGGTACCTGTGTGTGTGTGGGG |
| POX2m-F | GCATGCTCTAGAGTATACTTATATA |
| POX2-hrGFPO-R | AGG ATCTGCTTAGACACCATGGTACCGGCGTCGTTGCT |
| EXPm-F | GCATGCTCTAGAATTATATATAAGGC |
| TEF111-F | GCATGCTCTAGATATAAAAGACCACCGTCC |
| TEF136-F | GCATGCTCTAGATTGTGGTTGGGACT |
| TEF175-F | GCATGCTCTAGAATGCCGGACGCAAAATAG |
| TATA TEF-F | GCATGCTCTAGACACGGGCAAAAGTGCGTATAAAACAAGA |
| TATA EXP-F | GCATGCTCTAGACACGGGCAAAAGTGCGATTATATATAACAAGA |
| TATA PAT1-F | GCATGCTCTAGACACGGGCAAAAGTGCGTATATACCCAAGA |
| TATA POX2-F | GCATGCTCTAGACACGGGCAAAAGTGCGGTATACTTATATACAAGA |
| BDH-ADH2-F | CCATCCAGCCTCGCGTCGGTTAACTATCCTAGGGTGCATGCTGAG |
| BDH-ADH2-R | ACGTCTTGCTGGCGTTCGCGATCATCGATGATAAGCTGTCAAAC |
| BDH-ARO10-F | GCTTATCATCGATGATCGCGAAGTAGTAGGTTGAGGCCGTTG |
| BDH-ARO10-R | GCTACGTCTTGCTGGCGTTCGATGAGAATTCGGACACG |
| PEXP-ARO10-R | TCTCGATGGTCACGGGAGCCATCACGTGCTGTAGATATGT |
| POX2-ARO10-R | CGATGGTCACGGGAGCCATCACGTGGGCGTCGTTGCTTGTG |
| PTEF-ARO10-R | CTCGATGGTCACGGGAGCCATCACGTGTTTGAATGATTCTT |
| PLEU-ARO10-R | CTCGATGGTCACGGGAGCCATCACGTGTGGATGTGTGTGG |
| pYL-F | CCTCGATCCGGCATGCACTGATCACG |
| pYL-R | TAGGCAACAGCGTTGGGAGAGCCCTTGAGG |
| ADH2-CX-4-1 | CTACGAGAAGATGGAAAAGG |
| BAT1-YZ-R | ACCGCTCGTTGATGTCCCAC |
| CX-2 | TCTCCCTTATGCGACTCCTG |
| P-CX-R | CCCTTGGTCACTCTGATCTGC |
| ADH2-CX-F | CTACGAGAAGATGGAAAAGG |
| A-CX-R | CGGGCACGCCGAACACAGACTT |

**Table S2 Plasmids used in this study**

| Plasmid | Features | Reference |
| --- | --- | --- |
| pYLEX1 | *Y. lipolytica*‑integrative plasmid, P*_UAS1B4-LEUm_*-T_XPR2_, *LEU2* | [6] |
| pYLGFPuv | P*_UAS1B4-LEUm_*-*GFPuv*-T_XPR2_, *LEU2* | This study |
| pYLhrGFP | P*_UAS1B4-LEUm_*-*hrGFP*-T_XPR2_, *LEU2* | This study |
| pYLhrGFPO | P*_UAS1B4-LEUm_*-*hrGFPO*-T_XPR2_, *LEU2* | This study |
| pYLP*_LEU_* +hrGFPO | P*_LEU_*-*hrGFPO*-T_XPR2_, *LEU2* | This study |
| pYLP*_TEF_* +hrGFPO | P*_TEF_*-*hrGFPO*-T_XPR2_, *LEU2* | This study |
| pYLP*_EXP_*+hrGFPO | P*_EXP_*-*hrGFPO*-T_XPR2_, *LEU2* | This study |
| pYLP*_LEUm_*+hrGFPO | P*_LEUm_*-*hrGFPO*-T_XPR2_, *LEU2* | This study |
| pYLP*_UAS1B4-PAT1m_*+hrGFPO | P*_UAS1B4-PAT1m_*-*hrGFPO*-T_XPR2_, *LEU2* | This study |
| pYLP*_UAS1B4-POX2m_*+hrGFPO | P*_UAS1B4-POX2m_*-*hrGFPO*-T_XPR2_, *LEU2* | This study |
| pYLP*_UAS1B4-EXPm_*+hrGFPO | P*_UAS1B4-EXPm_*-*hrGFPO*-T_XPR2_, *LEU2* | This study |
| pYLP*_UAS1B4-TEF111_*+hrGFPO | P*_UAS1B4-TEF111_*-*hrGFPO*-T_XPR2_, *LEU2* | This study |
| pYLP*_UAS1B4-TEF136_*+hrGFPO | P*_UAS1B4-TEF136_*-*hrGFPO*-T_XPR2_, *LEU2* | This study |
| pYLP*_UAS1B4-TEF175_*+hrGFPO | P*_UAS1B4-TEF175_*-*hrGFPO*-T_XPR2_, *LEU2* | This study |
| pYLP*_UAS1B4-TATATEF-LEU_*+hrGFPO | P*_UAS1B4-TATATEF-LEU_*-*hrGFPO*-T_XPR2_, *LEU2* | This study |
| pYLP*_UAS1B4-TATAEXP-LEU_*+hrGFPO | P*_UAS1B4-TATAEXP-LEU_*-*hrGFPO*-T_XPR2_, *LEU2* | This study |
| pYLP*_UAS1B4-TATAPAT1-LEU_*+hrGFPO | P*_UAS1B4-TATAPAT1-LEU_*-*hrGFPO*-T_XPR2_, *LEU2* | This study |
| pYLP*_UAS1B4-TATAPOX2-LEU_*+hrGFPO | P*_UAS1B4-TATAPOX2-LEU_*-*hrGFPO*-T_XPR2_, *LEU2* | This study |
| pYLP*_UASCITSC4-LEUm_* +hrGFPO | P*_UASCITSC4-LEUm_*-*hrGFPO*-T_XPR2_, *LEU2* | This study |
| pYLP*_UASCLBSC4-LEUm_* +hrGFPO | P*_UASCLBSC4-LEUm_*-*hrGFPO*-T_XPR2_, *LEU2* | This study |
| pYLP*_UASTEFSC4-LEUm_* +hrGFPO | P*_UASTEFSC4-LEUm_*-*hrGFPO*-T_XPR2_, *LEU2* | This study |
| pYLP*_UASTEFYL4-LEUm_* +hrGFPO | P*_UASTEFYL4-LEUm_*-*hrGFPO*-T_XPR2_, *LEU2* | This study |
| pYLP*_UAS1B6-LEUm_*+hrGFPO | P*_UAS1B6-LEUm_*-*hrGFPO*-T_XPR2_, *LEU2* | This study |
| pYLP*_UAS1B8-LEUm_*+hrGFPO | P*_UAS1B8-LEUm_*-*hrGFPO*-T_XPR2_, *LEU2* | This study |
| pYLSCBAT1 | P*_UAS1B4-LEUm_*-*ScBAT1*-T_XPR2_, *LEU2* | This study |
| pYLSCARO10 | P*_UAS1B4-LEUm_*-*ScARO10*-T_XPR2_, *LEU2* | This study |
| pYLSCADH2 | P*_UAS1B4-LEUm_*-*ScADH2*-T_XPR2_, *LEU2* | This study |
| pYLYLBAT1-1 | P*_UAS1B4-LEUm_*-*YlBAT1-1*-T_XPR2_, *LEU2* | This study |
| pYLYLBAT1-2 | P*_UAS1B4-LEUm_*-*YlBAT1-2*-T_XPR2_, *LEU2* | This study |
| pYLYLARO10-1 | P*_UAS1B4-LEUm_*-*YlARO10-1*-T_XPR2_, *LEU2* | This study |
| pYLYLARO10-2 | P*_UAS1B4-LEUm_*-*YlARO10-2*-T_XPR2_, *LEU2* | This study |
| pYLYLADH2-1 | P*_UAS1B4-LEUm_*-*YlADH2-1*-T_XPR2_, *LEU2* | This study |
| pYLYLADH2-2 | P*_UAS1B4-LEUm_*-*YlADH2-2*-T_XPR2_, *LEU2* | This study |
| pYLYLADH2-3 | P*_UAS1B4-LEUm_*-*YlADH2-3-*T_XPR2_, *LEU2* | This study |
| pYLYLADH2-4 | P*_UAS1B4-LEUm_*-*YlADH2-4*-T_XPR2_, *LEU2* | This study |
| pYLYLADH2-5 | P*_UAS1B4-LEUm_*-*YlADH2-5*-T_XPR2_, *LEU2* | This study |
| pYLBAA | P*_UAS1B4-LEUm_*-*ScBAT1*-T_XPR2_, P*_UAS1B4-LEUm_*-*ScADH2*-T_XPR2_, P*_UAS1B4-LEUm_*-*ScARO10*-T_XPR2_, *LEU2* | This study |
| pYLBA+P*_EXP_*+ARO10 | P*_UAS1B4-LEUm_*-*ScBAT1*-T_XPR2_, P*_UAS1B4-LEUm_*-*ScADH2*-T_XPR2_, P*_EXP_*-*ScARO10*-T_XPR2_, *LEU2* | This study |
| pYLBA+P*_UAS1B4-EXPm_*+ARO10 | P*_UAS1B4-LEUm_*-*ScBAT1*-T_XPR2_, P*_UAS1B4-LEUm_*-*ScADH2*-T_XPR2_, P*_UAS1B4-EXPm_*-*ScARO10*-T_XPR2_, *LEU2* | This study |
| pYLBA+P*_UAS1B4-POX2m_*+ARO10 | P*_UAS1B4-LEUm_*-*ScBAT1*-T_XPR2_, P*_UAS1B4-LEUm_*-*ScADH2*-T_XPR2_, *_UAS1B4-POX2m_*-*ScARO10*-T_XPR2_, *LEU2* | This study |
| pYLBA+P*_UAS1B4-TEF136_*+ARO10 | P*_UAS1B4-LEUm_*-*ScBAT1*-T_XPR2_, P*_UAS1B4-LEUm_*-*ScADH2*-T_XPR2_, P*_UAS1B4-TEF136_*-*ScARO10*-T_XPR2_, *LEU2* | This study |
| pYLBA+P*_UASTEFLY4-LEUm_*+ARO10 | P*_UAS1B4-LEUm_*-*ScBAT1*-T_XPR2_, P*_UAS1B4-LEUm_*-*ScADH2*-T_XPR2_, P*_UASTEFLY4-LEUm_*-*ScARO10*-T_XPR2_, *LEU2* | This study |
| pYLBA+P*_UAS1B4-TATAEXP-LEU_*+ARO10 | P*_UAS1B4-LEUm_*-*ScBAT1*-T_XPR2_, P*_UAS1B4-LEUm_*-*ScADH2*-T_XPR2_, P*_UAS1B4-TATAEXP-LEU_*-*ScARO10*-T_XPR2_, *LEU2* | This study |
| pYLBA+P*_UAS1B4-TATATEF-LEU_*+ARO10 | P*_UAS1B4-LEUm_*-*ScBAT1*-T_XPR2_, P*_UAS1B4-LEUm_*-*ScADH2*-T_XPR2_, P*_UAS1B4-TATATEF-LEU_*-*ScARO10*-T_XPR2_, *LEU2* | This study |
| pYLBA+ P*_UAS1B8-LEUm_*+ARO10 | P*_UAS1B4-LEUm_*-*ScBAT1*-T_XPR2_, P*_UAS1B4-LEUm_*-*ScADH2*-T_XPR2_, P*_UAS1B8-LEUm_*-*ScARO10*-T_XPR2_, *LEU2* | This study |

**Table S3 Strains used in this study**

| Strains | Genotype | Reference |
| --- | --- | --- |
| Po1g *KU70*Δ | MatA, leu2-270, ura3-302::URA3, xpr2-332, axp-2, ku70- | [9] |
| Po1g P*_UAS1B4-LEUm_*+GFPuv | MatA, leu2-270, ura3-302::URA3, xpr2-332, axp-2, ku70-, P*_UAS1B4-LEUm_*-*GFPuv* | This study |
| Po1g P*_UAS1B4-LEUm_*+hrGFP | MatA, leu2-270, ura3-302::URA3, xpr2-332, axp-2, ku70-, P*_UAS1B4-LEUm_*-*hrGFP* | This study |
| Po1g P*_UAS1B4-LEUm_*+hrGFPO | MatA, leu2-270, ura3-302::URA3, xpr2-332, axp-2, ku70-, P*_UAS1B4-LEUm_*-*hrGFPO* | This study |
| Po1g P*_LEU_* +hrGFPO | MatA, leu2-270, ura3-302::URA3, xpr2-332, axp-2, ku70-, P*_LEU_*-*hrGFPO* | This study |
| Po1g P*_TEF_* +hrGFPO | MatA, leu2-270, ura3-302::URA3, xpr2-332, axp-2, ku70-, P*_TEF_*-*hrGFPO* | This study |
| Po1g P*_EXP_*+hrGFPO | MatA, leu2-270, ura3-302::URA3, xpr2-332, axp-2, ku70-, P*_EXP_*-*hrGFPO* | This study |
| Po1g P*_LEUm_*+hrGFPO | MatA, leu2-270, ura3-302::URA3, xpr2-332, axp-2, ku70-, P*_LEUm_*-*hrGFPO* | This study |
| Po1g P*_UAS1B4-PAT1m_*+hrGFPO | MatA, leu2-270, ura3-302::URA3, xpr2-332, axp-2, ku70-, P*_UAS1B4-PAT1m_*-*hrGFPO* | This study |
| Po1g P*_UAS1B4-POX2m_*+hrGFPO | MatA, leu2-270, ura3-302::URA3, xpr2-332, axp-2, ku70-, P*_UAS1B4-POX2m_*-*hrGFPO* | This study |
| Po1g P*_UAS1B4-EXPm_*+hrGFPO | MatA, leu2-270, ura3-302::URA3, xpr2-332, axp-2, ku70-, P*_UAS1B4-EXPm_*-*hrGFPO* | This study |
| Po1g P*_UAS1B4-TEF111_*+hrGFPO | MatA, leu2-270, ura3-302::URA3, xpr2-332, axp-2, ku70-, P*_UAS1B4-TEF111_*-*hrGFPO* | This study |
| Po1g P*_UAS1B4-TEF136_*+hrGFPO | MatA, leu2-270, ura3-302::URA3, xpr2-332, axp-2, ku70-, P*_UAS1B4-TEF136_*-*hrGFPO* | This study |
| Po1g P*_UAS1B4-TEF175_*+hrGFPO | MatA, leu2-270, ura3-302::URA3, xpr2-332, axp-2, ku70-, P*_UAS1B4-TEF175_*-*hrGFPO* | This study |
| Po1g P*_UAS1B4-TATATEF-LEU_*+hrGFPO | MatA, leu2-270, ura3-302::URA3, xpr2-332, axp-2, ku70-, P*_UAS1B4-TATATEF-LEU_*-*hrGFPO* | This study |
| Po1g P*_UAS1B4-TATAEXP-LEU_*+hrGFPO | MatA, leu2-270, ura3-302::URA3, xpr2-332, axp-2, ku70-, P*_UAS1B4-TATAEXP-LEU_*-*hrGFPO* | This study |
| Po1g P*_UAS1B4-TATAPAT1-LEU_*+hrGFPO | MatA, leu2-270, ura3-302::URA3, xpr2-332, axp-2, ku70-, P*_UAS1B4-TATAPAT1-LEU_*-*hrGFPO* | This study |
| Po1g *_UAS1B4-TATAPOX2-LEU_*+hrGFPO | MatA, leu2-270, ura3-302::URA3, xpr2-332, axp-2, ku70-, P*_UAS1B4-TATAPOX2-LEU_*-*hrGFPO* | This study |
| Po1g P*_UASCITSC4-LEUm_*+hrGFPO | MatA, leu2-270, ura3-302::URA3, xpr2-332, axp-2, ku70-, P*_UASCITSC4-LEUm_*-*hrGFPO* | This study |
| Po1g P*_UASCLBSC4-LEUm_*+hrGFPO | MatA, leu2-270, ura3-302::URA3, xpr2-332, axp-2, ku70-, P*_UASCLBSC4-LEUm_*-*hrGFPO* | This study |
| Po1g P*_UASTEFSC4-LEUm_*+hrGFPO | MatA, leu2-270, ura3-302::URA3, xpr2-332, axp-2, ku70-, P*_UASTEFSC4-LEUm_*-*hrGFPO* | This study |
| Po1g P*_UASTEFYL4-LEUm_*+hrGFPO | MatA, leu2-270, ura3-302::URA3, xpr2-332, axp-2, ku70-, P*_UASTEFYL4-LEUm_*-*hrGFPO* | This study |
| Po1g P*_UAS1B6-LEUm_*+hrGFPO | MatA, leu2-270, ura3-302::URA3, xpr2-332, axp-2, ku70-, P*_UAS1B6-LEUm_*-*hrGFPO* | This study |
| Po1g P*_UAS1B8-LEUm_*+hrGFPO | MatA, leu2-270, ura3-302::URA3, xpr2-332, axp-2, ku70-, P*_UAS1B8-LEUm_*-*hrGFPO* | This study |
| Po1g ScBAT1 | MatA, leu2-270, ura3-302::URA3, xpr2-332, axp-2, ku70-, P*_UAS1B4-LEUm_*-*SCBAT1* | This study |
| Po1g ScARO10 | MatA, leu2-270, ura3-302::URA3, xpr2-332, axp-2, ku70-, P*_UAS1B4-LEUm_*-*SCARO10* | This study |
| Po1g ScADH2 | MatA, leu2-270, ura3-302::URA3, xpr2-332, axp-2, ku70-, P*_UAS1B4-LEUm_*-*SCADH2* | This study |
| Po1g YlBAT1-1 | MatA, leu2-270, ura3-302::URA3, xpr2-332, axp-2, ku70-, P*_UAS1B4-LEUm_*-*YlBAT1-1* | This study |
| Po1g YlBAT1-2 | MatA, leu2-270, ura3-302::URA3, xpr2-332, axp-2, ku70-, P*_UAS1B4-LEUm_*-*YlBAT1-2* | This study |
| Po1g YlARO10-1 | MatA, leu2-270, ura3-302::URA3, xpr2-332, axp-2, ku70-, P*_UAS1B4-LEUm_*-*YlARO10-1* | This study |
| Po1g YlARO10-2 | MatA, leu2-270, ura3-302::URA3, xpr2-332, axp-2, ku70-, P*_UAS1B4-LEUm_*-*YlARO10-2* | This study |
| Po1g YlADH2-1 | MatA, leu2-270, ura3-302::URA3, xpr2-332, axp-2, ku70-, P*_UAS1B4-LEUm_*-*YlADH2-1* | This study |
| Po1g YlADH2-2 | MatA, leu2-270, ura3-302::URA3, xpr2-332, axp-2, ku70-, P*_UAS1B4-LEUm_*-*YlADH2-2* | This study |
| Po1g YlADH2-3 | MatA, leu2-270, ura3-302::URA3, xpr2-332, axp-2, ku70-, P*_UAS1B4-LEUm_*-*YlADH2-3* | This study |
| Po1g YlADH2-4 | MatA, leu2-270, ura3-302::URA3, xpr2-332, axp-2, ku70-, P*_UAS1B4-LEUm_*-*YlADH2-4* | This study |
| Po1g YlADH2-5 | MatA, leu2-270, ura3-302::URA3, xpr2-332, axp-2, ku70-, P*_UAS1B4-LEUm_*-*YlADH2-5* | This study |
| Po1g BAA | MatA, leu2-270, ura3-302::URA3, xpr2-332, axp-2, ku70-, P*_UAS1B4-LEUm_*-*ScBAT1*, P*_UAS1B4-LEUm_*-*ScADH2*, P*_UAS1B4-LEUm_*-*ScARO10* | This study |
| Po1g BA+P*_EXP_*+ARO10 | MatA, leu2-270, ura3-302::URA3, xpr2-332, axp-2, ku70-, P*_UAS1B4-LEUm_*-*ScBAT1*, P*_UAS1B4-LEUm_*-*ScADH2*, P*_EXP_*-*ScARO10* | This study |
| Po1g BA+P*_UAS1B4-EXPm_*+ARO10 | MatA, leu2-270, ura3-302::URA3, xpr2-332, axp-2, ku70-, P*_UAS1B4-LEUm_*-*ScBAT1*, P*_UAS1B4-LEUm_*-*ScADH2*, P*_UAS1B4-EXPm_*-*ScARO10* | This study |
| Po1g BA+P*_UAS1B4-POX2m_*+ARO10 | MatA, leu2-270, ura3-302::URA3, xpr2-332, axp-2, ku70-, P*_UAS1B4-LEUm_*-*ScBAT1*, P*_UAS1B4-LEUm_*-*ScADH2*, P*_UAS1B4-POX2m_*-*S_C_ARO10* | This study |
| Po1g BA+P*_UAS1B4-TEF136_*+ARO10 | MatA, leu2-270, ura3-302::URA3, xpr2-332, axp-2, ku70-, P*_UAS1B4-LEUm_*-*S_C_BAT1*, P*_UAS1B4-LEUm_*-*S_C_ADH2*, P*_UAS1B4-TEF136_*-*ScARO10* | This study |
| Po1g BA+P*_UASTEFLY4-LEUm_*+ARO10 | MatA, leu2-270, ura3-302::URA3, xpr2-332, axp-2, ku70-, P*_UAS1B4-LEUm_*-*ScBAT1*, P*_UAS1B4-LEUm_*-*ScADH2*, P*_UASTEFLY4-LEUm_*-*ScARO10* | This study |
| Po1g BA+P*_UAS1B4-TATAEXP-LEU_*+ARO10 | MatA, leu2-270, ura3-302::URA3, xpr2-332, axp-2, ku70-, P*_UAS1B4-LEUm_*-*ScBAT1*, P*_UAS1B4-LEUm_*-*ScADH2*, P*_UAS1B4-TATAEXP-LEU_*-*ScARO10* | This study |
| Po1g BA+P*_UAS1B4-TATATEF-LEU_*+ARO10 | MatA, leu2-270, ura3-302::URA3, xpr2-332, axp-2, ku70-, P*_UAS1B4-LEUm_*-*ScBAT1*, P*_UAS1B4-LEUm_*-*ScADH2*, P*_UAS1B4-TATATEF-LEU_*-*ScARO10* | This study |
| Po1g BA+P*_UAS1B8-LEUm_*+ARO10 | MatA, leu2-270, ura3-302::URA3, xpr2-332, axp-2, ku70-, P*_UAS1B4-LEUm_*-*ScBAT1*, P*_UAS1B4-LEUm_*-*ScADH2*, P*_UAS1B8-LEUm_*-*ScARO10* | This study |

**Sequence of all core promoters**

LEU core promoter sequence:

TATATATACAAGAGCGTTTGCCAGCCACAGATTTTCACTCCACACACCACATCACACATACAACCACACACATCCACA

EXP core promoter sequence:

ATTATATATAAGGCTCGTCTCTCCCTCCCAACCACACTCACTTTTTTGCCCGTCTTCCCTTGCTAACACAAAAGTCAAGAACACAAACAACCACCCCAACCCCCTTACACACAAGACATATCTACAGCA

PAT1 core promoter sequence:

TATATACCTCGGTCCTTCTCTTTCTGCCATCCACGTCACATATCCTCCCCACACACACACA

POX2 core promoter sequence:

GTATACTTATATACCAAAGGGATGGGTCCTCAAAAATCACACAAGCAACGACGCC

TEF111 core promoter sequence:

TATAAAAGACCACCGTCCCCGAATTACCTTTCCTCTTCTTTTCTCTCTCTCCTTGTCAACTCACACCCGAAATCGTTAAGCATTTCCTTCTGAGTATAAGAATCATTCAAA

TEF136 core promoter sequence:

TTGTGGTTGGGACTTTAGCCAAGGGTATAAAAGACCACCGTCCCCGAATTACCTTTCCTCTTCTTTTCTCTCTCTCCTTGTCAACTCACACCCGAAATCGTTAAGCATTTCCTTCTGAGTATAAGAATCATTCAAA

TEF175 core promoter sequence:

ATGCCGGACGCAAAATAGACTACTGAAAATTTTTTTGCTTTGTGGTTGGGACTTTAGCCAAGGGTATAAAAGACCACCGTCCCCGAATTACCTTTCCTCTTCTTTTCTCTCTCTCCTTGTCAACTCACACCCGAAATCGTTAAGCATTTCCTTCTGAGTATAAGAATCATTCAAA

**References:**

1. Blazeck J, Liu LQ, Redden H, Alper HS. Tuning gene expression in *Yarrowia lipolytica* by a hybrid promoter approach. Appl Environ Microbiol. 2011;77(22):7905-7914.

2. Hong SP, Seip J, Walters-Pollak D, Rupert R, Jackson R, Xue Z, Zhu Q. Engineering *Yarrowia lipolytica* to express secretory invertase with strong FBA1IN promoter. Yeast. 2012;29(2):59-72.

3. Shabbir Hussain M, Gambill L, Smith S, Blenner MA. Engineering promoter architecture in oleaginous yeast *Yarrowia lipolytica*. ACS Synth Biol. 2016;5(3):213-223.

4. Blazeck J, Garg R, Reed B, Alper HS. Controlling promoter strength and regulation in *Saccharomyces cerevisiae* using synthetic hybrid promoters. Biotechnol Bioeng. 2012;109(11):2884-2895.

5. Hicks BW. Green fluorescent protein: applications and protocols. Humana Press 2002. p. 35-40.

6. Madzak C, Tréton B, Blanchin-Roland S. Strong hybrid promoters and integrative expression/secretion vectors for quasi-constitutive expression of heterologous proteins in the yeast *Yarrowia lipolytica*. J Mol Microbiol Biotechnol. 2000;2(2):207-216.

7. Toh YH, Huang YW, Chang YC, Chen YT, Hsu YT, Lin GH. Reactivity of human antisera to codon optimized SARS-CoV2 viral proteins expressed in *Escherichia coli*. Tzu Chi Med J. 2021;33(2):146-153.

8. Wang Z, Xu B, Li B, et al. Comparative analysis of codon usage patterns in chloroplast genomes of six *Euphorbiaceae* species. PeerJ. 2020;8:e8251.

9. Yu AQ, Pratomo N, Ng TK, Ling H, Cho HS, Leong SS, Chang MW. Genetic engineering of an unconventional yeast for renewable biofuel and biochemical production. J Vis Exp. 2016;20(115):54371.
